# Effectiveness of Obliterative Surgery in Managing Advanced Apical Prolapse in Elderly Women: A 20-Years of Single Surgeon’s Experience

**DOI:** 10.3390/jcm14093101

**Published:** 2025-04-30

**Authors:** Dayong Lee, Tae Hun Kim, Taek Sang Lee

**Affiliations:** Department of Obstetrics and Gynecology, SMG-SNU Boramae Medical Center, Seoul 07061, Republic of Korea; wabhappy@gmail.com

**Keywords:** pelvic organ prolapse, colpocleisis, elderly women

## Abstract

**Objectives:** The aim of this study was to evaluate the surgical outcomes, therapeutic efficacy, and psychological satisfaction of the Le Fort partial colpocleisis (LFC) procedure, an obliterative surgical treatment option, in elderly women with advanced apical prolapse, based on the 20-year surgical experience of a single surgeon. **Methods:** A retrospective cohort study was conducted on 81 women aged 60 and older who underwent LFC for advanced apical prolapse at a single institution from 2006 to 2025. Baseline characteristics, comorbidities, perioperative outcomes, complications, and patients’ satisfaction were analyzed. **Results:** Among the patients, 86.4% were aged 70 or older, and also 85.2% of the women had comorbidities that could influence surgical outcomes. The surgical success rate was 96.3%, with recurrences observed in three cases. The median operative time was 89 min, but it decreased to median 77 min as the accumulated surgeon’s experience. Similarly, hospitalization duration and patient-reported postoperative pain score also showed significant reductions. Transient postoperative complications were minimal, predominantly transient urinary symptoms (voiding difficulty in 4.9%, urinary frequency in 6.2%, and urinary incontinence in 3.7% of the patients). Satisfaction with the surgical treatment was high, with 98.4% reporting overall satisfaction. **Conclusion:** LFC is a safe and effective option for elderly women with advanced apical prolapse, offering high satisfaction rates and low morbidity. Surgeon’s experience significantly enhances outcomes after about 20 cases. Careful patient selection and throughout counseling are essential to optimize outcomes and address patients’ satisfaction by enhancing physical and psychological quality of life.

## 1. Introduction

The vaginal canal is anatomically surrounded by the bladder, uterus, and rectum, with these organs supported and stabilized by a complex structure of connective tissues and muscles. A defect in these supporting structures of the vagina can lead to the descent of the anterior or posterior vaginal wall, the uterus, or the vaginal stump after hysterectomy, a condition collectively referred to as pelvic organ prolapse (POP) [1]. The primary risk factors for POP include parity, vaginal delivery, age, and body mass index (BMI) [2]. As fertility rates decline in developed countries, the incidence of POP due to increased parity is expected to decrease. However, the aging population will likely lead to a rise in age-related POP cases, emphasizing the need for effective management of POP in the elderly population in the future. POP negatively impacts women’s social, physical, and psychological well-being [3]. Consequently, as the overall population ages, POP is projected to become a significant factor in diminishing women’s quality of life and restricting their social relationship and participation, thereby imposing both personal and societal burdens.

According to the United Nations (UN) criteria, population aging is assessed based on the proportion of individuals aged 65 years or older within the total population. The global age distribution published by the UN indicates that the proportion of individuals aged 65 years or older is projected to increase from 10.2% in 2024 to approximately 20.3% by 2072, nearly doubling [4]. Similarly, a report by Statistics Korea predicts that the proportion of the elderly population in South Korea will sharply rise from 19.2% in 2024 to 47.7% by 2072 [5]. This exaggerated tendency is attributed to the continuous increase in life expectancy in South Korea, coupled with the country’s worldwide record-low fertility rate. As societal aging progresses, the importance of managing age-related diseases is becoming increasingly significant. Among these aging-related conditions, there is growing attention to pelvic floor disorders, including POP.

The treatment of POP is determined based on the degree of prolapse and the severity of associated symptoms. For mild to moderate prolapse, conservative management options such as pelvic floor muscle training and biofeedback can be employed. However, in more severe cases, interventions such as pessary insertion or surgical treatment are required. Surgical treatment for POP is grossly categorized into reconstructive and obliterative procedures. Reconstructive techniques aim to preserve vaginal depth and functionality. However, in older women who no longer engage in sexual activity, and those at higher risk of complications from surgery or women with significant underlying comorbidities, obliterative procedures serve as an effective alternative for correcting advanced apical prolapse. Le Fort partial colpocleisis (LFC) is a representative obliterative procedure that can be applied to older, frail women with advanced apical prolapse. While colpocleisis offers an effective solution, it carries significant drawbacks, including a potential negative impact on a woman’s self-body image and the permanent inability to engage in sexual activity. Therefore, careful patient selection and counseling are crucial before determining the procedure. With the progression of global aging, the number of elderly women requiring surgical intervention for POP is expected to increase, highlighting the growing relevance of this procedure. To date, LFC remains one of the least invasive surgical option for POP, offering a short recovery period while being highly effective and associated with low perioperative morbidity [6]. Nevertheless, reports on LFC remain limited, with most studies being small case series involving a restricted number of patients. These studies often lack comprehensive outcome analysis or adequate follow-up. This study aims to analyze the surgical outcomes, postoperative complications, patient satisfaction, and regret rates of LFC for women with advanced apical prolapse, based on the 20-year experience of a single surgeon. Analyzing the outcomes over an extended period when a single physician consults patient and performs treatments using consistent surgical criteria and techniques will provide meaningful data to evaluate the utility and safety of this surgical method.

## 2. Materials and Methods

### 2.1. Study Design and Database Population

This study was performed as a retrospective cohort study, targeting women who underwent the LFC procedure for advanced apical prolapse. Data were collected from January 2006 to February 2025, encompassing cases performed by a single surgeon (T.S. Lee) at the Seoul Metropolitan Government–Seoul National University Boramae Medical Center. Surgery was performed only in women who consented to surgical treatment for pelvic organ prolapse and who did not have any medical, physical, or psychological conditions that would preclude surgery.

The severity of apical prolapse in the study population was assessed using the Pelvic Organ Prolapse Quantification (POP-Q) system, introduced in 1996 [7]. For POP-Q scoring, patients were examined in a standing position with legs shoulder-width apart and knees slightly bent to allow maximal prolapse manifestation. The surgeon performed the evaluation while the patient applied downward pressure to maximize pelvic organ bulging. Surgery was performed for patients with a completely prolapsed uterus (stage IV) who reported symptoms and discomfort caused by the condition. Preoperative ultrasound examinations and Pap smear results were acquired to rule out the possibility of associated malignancies.

Baseline patient characteristics, co-morbidities, and prolapse severity were collected through a review of electronic medical records for women who underwent surgery. Perioperative and postoperative data, including complications, were also analyzed. Preoperative comorbidities were categorized into eight groups; (1) cardiovascular disease and its risk factors, including hypertension, dyslipidemia, cardiomegaly, angina, and arrhythmia, (2) pulmonary disease, including history of tuberculosis, asthma, and chronic obstructive pulmonary disease, (3) diabetes, (4) thyroid disease, (5) liver disease, including hepatitis and liver cirrhosis, (6) renal disease, (7) history of non-gynecologic cancer, (8) neurologic disease, including history of stroke, subarachnoid hemorrhage, cerebrovascular attack, and spinal disease, and (9) neuropsychiatric disease, including depression and dementia. Surgical data were collected including the total operating time (from incision to closure), types of intraoperative events, and the postoperative hemoglobin (Hb) drop. Hb drops were measured routinely for all patients through blood sampling on the day after surgery. Postoperative data included the duration of hospitalization in days, postoperative pain, and the occurrence of perioperative complications. Postoperative pain was measured using the numerical rating scale (NRS). Pain levels were assessed and recorded on the mornings of postoperative day 1 and day 2. A score of 0 represented no pain at all, while a score of 10 indicated the most severe pain imaginable. Perioperative complications were defined as those occurring within three months after surgery. Surgical success was defined as the absence of vaginal wall protrusion over vaginal hymen (POP-Q stage ≤ 1) [2]. Additionally, patient satisfaction was assessed through interviews conducted during outpatient visits or via telephone contacts. In cases where direct consultation with the patient was challenging due to their condition, information about the patient’s expressed satisfaction after surgery was collected through their family members or caregivers. The following questions were asked: (1) Compared to your condition before surgery, how satisfied are you with the current surgical outcome? Patient satisfaction was categorized as “very satisfied” “satisfied despite some discomfort compared to pre-surgery,” or “regret.” (2) If you are dissatisfied with the surgical outcome, what is the primary reason? Cases where patients could not be reached for follow-up were recorded as missing data. The study was conducted with the approval of the SMG-SNU Boramae Medical Center Institutional Review Board (IRB) (IRB approval no. 10-2018-83) and in compliance with both national and international guidelines, including the principles outlined in the Declaration of Helsinki. As no personally identifiable information was collected and the study procedures had no impact on the participants, the requirement for informed consent was waived.

### 2.2. Surgical Procedure

The LFC surgical procedure was conducted according to the method previously described by the research team [8]. Briefly, the epithelial tissue of the anterior and posterior vaginal walls was carefully dissected into thin rectangular strips. The internal edges of each distal rectangle were sutured together, and the denuded areas were aligned and sewn using 2-0 Vicryl interrupted sutures from anterior to posterior. This approach repositioned the bulging apical prolapse inside the vaginal cavity while preserving the lateral margins of the vaginal canal. Additionally, if indicated, levator ani plication and posterior perineorrhaphy were performed to secure the apical lesion to the pelvic floor.

### 2.3. Statistical Analysis

The data were analyzed using SPSS version 20.0 (SPSS Inc.; Chicago, IL, USA). Continuous variables were expressed as mean ± standard deviation for normally distributed data and as median [range] for non-normally distributed data. Categorical variables were presented as frequency (percentage). Comparisons between two independent groups were performed using the Student’s *t*-test for normally distributed data and the Mann–Whitney U test for non-normally distributed data. The chi-square test was used to compare proportions between two groups. When more than 20% of the cells had an expected frequency of less than 5, Fisher’s exact test was applied. A *p*-value of <0.05 was considered statistically significant.

## 3. Results

During the study period, the LFC procedure for stage IV advanced apical prolapse was planned for 83 women. However, in two cases, muscle rigidity occurred after spinal anesthesia, preventing sufficient descent of the prolapsed area and making adequate surgical exposure impossible. As a result, these two cases were converted to vaginal total hysterectomy. Consequently, a total of 81 women underwent the LFC procedure. Among them, 17 (21.0%) women had a history of prior hysterectomy. In one case, the cervix was too large to accommodate the anterior and posterior denuded vaginal surfaces, necessitating vaginal total hysterectomy followed by colpocleisis. Among all surgical cases, concurrent posterior colporrhaphy was performed in 26 (32.1%) cases. All participants were aged 60 or older, with 86.4% being over 70 years old, indicating that the majority of the women were of advanced age.

Other clinical characteristics of the patients are summarized in Table 1. Among these women, 14 (17.3%) had prior experience using a pessary, and most of them decided surgical treatment due to recurrent pessary expulsion. Additionally, 85.2% of the women had at least one comorbidity. The most prevalent comorbidity was cardiovascular diseases, with 74.1% of women affected, followed by diabetes, which was present in 22.2% of women. The median interval from the onset of POP symptoms to the first outpatient clinic visit for treatment was 44.5 months, with a range from 1 to 360 months, indicating significant variability in the time taken by women to seek medical care after symptom onset.

Regarding surgical data, prolapse recurrence was observed in three cases, yielding a surgical success rate of 96.3%. Among these, one case involved a patient with accompanying constipation, resulting in the detachment of the previously sewn surface 11 months after surgery. The patient underwent re-operation after the application of topical estrogen cream to the vaginal wall to induce re-epithelialization, and no further recurrence was observed. The remaining two cases involved recurrent rectocele. As the patients did not report significant discomfort from their symptoms, these cases are currently being managed with observation rather than further surgical intervention.

**Figure 1:**
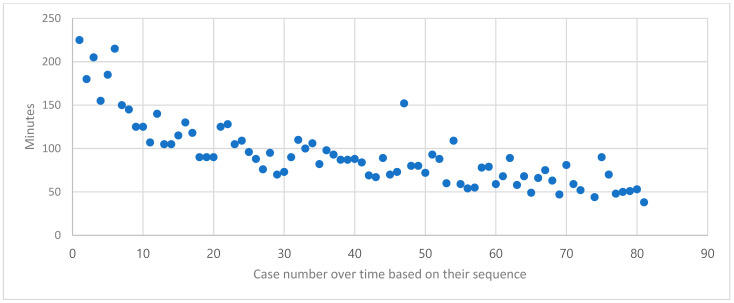
Duration of surgery based on order in which surgeries were performed.

Since the data were analyzed based on the chronological experience of a single surgeon, the impact of surgical experience on the outcomes could be assessed. Surgical duration is one of the most closely associated indicators of a surgeon’s experience. Therefore, the surgical times were analyzed sequentially and presented in Figure 1. A consistent decrease in surgical duration was observed over time, with stable operating times achieved after the initial 20 cases. Based on this observation, the postoperative outcomes of the first 20 surgeries were compared with those of the subsequent 61 surgeries, as shown in Table 2.

The median hospital stay was 2 days, with a significant difference observed between earlier and more recent surgeries, showing a median of 3 days versus 2 days, respectively. Except for one case with a 9-day hospital stay, all cases had a hospital stay of 4 days or less. The patient with a 9-day stay had preoperative thrombocytopenia, requiring collaborative care with internal medicine and surgery teams, which delayed discharge. Even when this case was excluded from the analysis, the trend of shorter hospital stays in later surgeries remained unchanged. The median hemoglobin (Hb) decrease following surgery was 1.7, with an average reduction of 14.8%. No significant difference in Hb reduction was observed between the early and later surgical cases. The median operative time was 89 min [range: 38–225]. For the initial 20 cases, the median operative time was 127.5 min [range: 90–225], whereas for the subsequent 61 cases, it decreased to 77 min [range: 38–152]. Most surgeries were completed within 150 min after the initial 20 cases, and the operative time continued to decrease even beyond the first 20 procedures. The median pain score on the NRS was 3 on postoperative day 1 and remained at 3 on day 2, indicating consistently low levels of postoperative pain. Moreover, the patients’ perceived pain significantly decreased as the surgeon gained more experience, reflecting an improvement in postoperative outcomes.

The most common transient postoperative complications were urinary symptoms, with voiding difficulty occurring in four cases (4.9%), urinary frequency in five cases (6.2%), and urinary incontinence in three cases (3.7%) (Table 3). Other complications included wound infection, vaginitis, constipation, and perineal sensory abnormalities, each reported in one case. All of these complications resolved over time. One case of suture site laceration was reported, which required revision surgery for reinforcement. No significant differences in the incidence of transient complications were observed between the early and later surgeries. Regarding long-term complications, one case of persistent urinary incontinence was observed, and two cases of de novo rectal prolapse occurred, both of which were managed by the relevant department.

Postoperative satisfaction was assessed in 64 women. Among them, 84.4% reported being “very satisfied” with no discomfort, while 14.1% stated they were satisfied compared to their preoperative condition. Overall, 63 women (98.5%) expressed satisfaction with the surgery. One woman, who did not report satisfaction, underwent surgery in 2009 and later experienced a de novo rectal prolapse. In 2014, she underwent sigmoidectomy and rectopexy to address the issue.

## 4. Discussion

This study demonstrated that the LFC procedure is an effective and safe surgical option for managing advanced apical prolapse in highly elderly women with multiple comorbidities that could potentially impact surgical outcomes. Furthermore, the result confirmed that once a certain level of surgical experience is attained, the procedure can consistently yield favorable outcomes. To the best of our knowledge, this study is the first to objectively compare the outcomes of an adequate number of LFC procedures performed by a single surgeon over an extended period, specifically analyzing and comparing surgical outcomes in relation to the surgeon’s accumulation of experience over time.

Pessary therapy and surgical treatment are both effective options for managing POP. Given that pessary therapy is a minimally invasive approach, physicians often deliberate on which treatment to choose. To address this question, Lisa et al. conducted a randomized controlled trial (RCT) comparing the effects of pessary therapy and surgical treatment in symptomatic POP patients [9]. The study, which involved 440 participants, reported that an initial strategy of pessary therapy met the criteria for non-inferiority compared to surgical treatment after 24 months. However, it is notable that in this study, crossover from pessary therapy to surgery occurred in a high proportion of women (54.1%, 118/218). Additionally, 42.7% of pessary users reported discomfort, and 60% discontinued pessary use within 24 months. A recent systematic review analyzed a total of seven studies and found that the pessary discontinuation rate ranged relatively high from 37% to 80% (median 49.1%). Key factors leading to failure in retaining the pessary included discomfort or pain, expulsion of the device, and difficulties in self-inserting or removing the pessary [10]. Furthermore, pessary users reported complications such as vaginal discharge, infections, and ulcers in the vaginal mucosa caused by contact with the device. A prospective study that observed 236 women over 12 months reported vulvovaginitis in 44.5% of cases, followed by vaginal ulceration in 16.4% [11]. Maintaining pessaries becomes more challenging for elderly women due to difficulties in managing perineal hygiene and periodically removing and cleaning the device. With these factors, the risk of complications caused by infections rises with age. A retrospective study analyzing 15 years of long-term data from 2007 to 2022 on women who used pessaries revealed a median duration of pessary retention of 3.3 years [12]. Approximately 50% of women maintained the pessary during the observation period. However, about 30% ultimately opted for surgical treatment, with most of these decisions made within four months of starting pessary use. This evidence highlights that while some women are able to use pessaries successfully over the long term, a substantial proportion eventually require surgical treatment due to discomfort or complications. Therefore, it is essential to provide surgical treatment as an option at an appropriate time for those who experience symptomatic POP.

According to a survey about practice patterns of colpocleisis conducted among members of the American Urogynecologic Society, responses were received from 322 out of 1422 clinicians (23%) [13]. Among the respondents, 82% identified as urogynecologists. Of these, the majority (97%) reported performing colpocleisis as part of their clinical practice. The top three reasons cited for conducting colpocleisis were its high success rate, short operating time, and low complication rates, in the order. Many studies reporting success rates after partial or total colpocleisis show success rates ranging from 91% to 100% [14]. However, many of these studies are over 30 years old and do not reflect the advancements in modern surgical practices, and the definition of recurrence varies across studies leading to various limitations. In our study, there were three cases (3.7%) of POP recurrence, which is similar with the recurrence rates reported in previous studies. According to a prospective multi-center study conducted by FitzGerald et al., the rate of maintaining a POP stage of ≤1 one year after surgery was 73%. [15]. In the current study, the recurrences were observed at 4 months, 11 months, and 16 months after surgery. In our observation, de novo rectal prolapse occurred 7 and 60 months after LFC. The occurrence of de novo rectal prolapse after advanced POP repair with colpocleisis and levator ani plication has been reported in case reports and case series [16]. In a previous study analyzing 92 cases over 12 years, two cases of rectal prolapse were reported [17]. With overt POP, the endopelvic connective tissue defect is not confined to a focal area but more likely affects various structures within the pelvic floor. Colpocleisis with levator ani plication utilizes the dysfunctional pelvic floor musculature as a physical barrier to visceral organ descent, narrowing the levator hiatus. Thus, repairing or supporting one part of the pelvic floor structure has been theorized to increase pressure on other areas, potentially provoking de novo defects in those regions [18]. This suggests that additional recurrence cases can arise with longer follow-up periods in other cases. Therefore, to accurately assess the success rate of the surgery, long-term follow-up with objective outcome monitoring would be necessary.

From as early as 1867, Neugebauer performed colpocleisis and published his results in 1881. In 1877, Le Fort introduced the techniques that underpin current modern practices, establishing colpocleisis as a long-standing surgical procedure [14]. Nevertheless, most studies reporting the outcomes of this procedure involved only a small number of cases. For instance, in 2004, O’Leary et al. reported the outcomes of 27 cases of LFC performed by a single surgeon over seven years [19]. The median operating time was 35 min [range 30–60], and to reduce operating time, the surgeon approximated the vaginal skin edges using continuous suturing with four or five bites rather than interrupted sutures. The success rate was reported as 89%, and at three months post-surgery, 100% of patients expressed satisfaction for their outcomes. However, that study analyzed surgical outcomes with a relatively small patient population, and thus could not evaluate differences in outcomes based on the accumulation of experience by a single surgeon. The relatively short median operating time of 35 min in the study likely reflected the absence of additional supporting procedures such as levator ani plication and posterior perineorrhaphy which were routinely performed in our procedure protocol for LFC. More recently, Keskin et al. reported outcomes of 64 cases of LFC performed by multiple surgeons over seven years at a university hospital. In their study, the mean operating time was 70.9 ± 24.6 min ranging 40 to 130 min [20]. In the present study, the mean operating time for the initial 20 cases was 127.5 min, which significantly decreased to median 77 min in subsequent cases. This reduction highlights the impact of the surgeon’s accumulated experience, which not only shortened operating times but also significantly decreased hospitalization durations as well as postoperative pain. Despite the differences in outcomes between early and later cases, there was no significant difference in perioperative complications, reaffirming that LFC is a safe procedure for elderly patients.

In this study, no intraoperative complications occurred among the cases. Transient postoperative complications were also minimal, with no severe complications observed. Most issues were transient urinary symptoms. Women undergoing surgery for POP often develop de novo stress urinary incontinence (SUI) postoperatively. While SUI may coexist with POP, the symptoms might not manifest before prolapse correction, a condition referred to as occult SUI. This phenomenon is attributed to urethral kinking and bladder outlet obstruction caused by the severe vaginal prolapse, which mask the incontinence [21]. In previous surveys, fourteen percent of urogynecology specialists who answered recommended an anti-incontinence procedure for all patients undergoing colpocleisis [13]. However, in this study, the risk of SUI after LFC was low, and most cases improved spontaneously. Therefore, performing the procedure simultaneously requires not only careful risk prediction but also consideration of the potential complications associated with the anti-incontinence procedure. Side effects other than urinary symptoms occurred much less frequently. One case of perineal wound infection was successfully treated with additional antibiotics, and another case of suture site laceration, caused by a sudden increase in intra-abdominal pressure due to abrupt severe sneezing, was repaired without further complications. Advanced age is a significant factor associated with postoperative complications in urogynecologic surgeries, with cardiovascular, respiratory, endocrine, renal, and neuropsychiatric conditions all contributing to increased morbidity and mortality risks. However, for obliterative procedures to treat POP, the incidence of complications is reported to be significantly lower in women aged 80 and older compared to reconstructive procedures [22]. In this study, 50% of the included women were in their 70s, and 36.8% were aged 80 or older. Despite the presence of various comorbidities in most participants, the rates of intraoperative and postoperative complications were remarkably low.

Satisfaction levels among women undergoing LFC procedures have been reported to be consistently high in previous studies. Song et al. documented a satisfaction rate of 94.3% among 35 women who underwent LFC [23]. In a study by Zebede et al. that followed a large group of 310 elderly women, 92.9% of the participants expressed satisfaction with their surgical outcomes [24]. In the present study, apart from one case of rapid POP recurrence, the majority of women reported satisfaction with the surgical results. Concerns regarding obliterative procedures such as their impact on sexual activity or potential negative effects on body image should not deter clinicians from referring patients to specialists who can perform these surgeries. The results of this study show that many women delay seeking medical help for years after experiencing POP symptoms. POP not only interferes with physical activity but also significantly impacts social relationships and self-image. Therefore, it is crucial to ensure these women receive the necessary treatment. By carefully selecting surgical candidates and providing thorough explanations about the procedure, the likelihood of achieving positive outcomes and high satisfaction would be possible.

There are some limitations in this study. First, this is the retrospective case series study which imposes restrictions on data collection regarding surgical parameters and postoperative outcomes. For the same reason, the duration of postoperative follow-up varied among patients, which may have introduced bias into the results. Additionally, there were significant variations in follow-up durations among patients. Second, patient satisfaction was assessed using a simplified survey. While some assessments were conducted during outpatient visits, many cases relied on telephone interviews, limiting the scope for evaluating various aspects of postoperative satisfaction. In addition, most of the women included in the study were of advanced age, and for many of them, several years had passed since their surgery. Therefore, considering the condition of the participants, only a minimal survey focusing on key aspects could be conducted.

The strength of this study lies in the data collection by a single surgeon who treated patients consistently over an extended period. This allowed for the accumulation of data based on uniform patient selection criteria and surgical protocols. To date, no study has analyzed the experience of a single surgeon spanning about 20 years and involving more than 80 cases. With this advantage, the current study highlights how accumulated surgical experience contributes to improved outcomes of LFC over time.

In conclusion, LFC proves to be an effective treatment option for POP in elderly women with multiple comorbidities. This procedure is characterized by its short operative time, minimal postoperative pain, rapid recovery, low complication rates, and high patient satisfaction. Notably, sufficient expertise in LFC can be achieved after approximately 20 cases, and consistent, stable, and safe outcomes can be expected thereafter. Therefore, with careful patient selection and thorough preoperative explanation and consent, the procedure can achieve high postoperative satisfaction. It can also contribute to improvements in daily functioning and psychological well-being, ultimately enhancing the quality of life for these women.

## Figures and Tables

**Table 1:** Characteristics of patients (n = 81).

Characteristics		Number of Patients (%)
Age at operation	60–69	11 (13.6%)
	70–79	41 (50.6%)
	80–89	28 (34.6%)
	90–99	1 (1.2%)
	Median [range]	76 [61–91]
Parity	1	4 (4.9%)
	2	10 (12.3%)
	3	25 (30.9%)
	4	37 (45.7%)
	5	5 (6.2%)
Previous pessary insertion		14 (17.3%)
Previous hysterectomy		17 (21.0%)
Type of comorbidity	Cardiovascular disease	60 (74.1%)
	Pulmonary disease	5 (6.2%)
	Diabetes	18 (22.2%)
	Thyroid disease	3 (3.7%)
	Liver disease	1 (1.2%)
	Renal disease	3 (3.7%)
	History of non-gynecologic cancer	1 (1.2%)
	Neurologic disease	13 (16.0%)
	Neuropsychiatric disease	11 (13.6%)
Number of comorbidities	0	12 (14.8%)
	1	29 (35.8%)
	2	25 (30.9%)
	3	10 (12.3%)
	4	4 (4.9%)
	5	1 (1.2%)
Duration from symptom onset to hospital visit (months)	Median [range]	44.5 [1–360]

**Table 2:** Operative outcomes (n = 81).

Characteristics	Total Cases	Initial 20 Cases	Subsequent 61 Cases	*p* Value
Hospitalization days	2 [1–9]	3 [1–9]	2 [1–4]	0.003
Hemoglobin decrease (mg/dL)	1.7 [0–4.8]	1.8 [0–4.8]	1.7 [0–3.6]	NS
Hemoglobin decrease (%)	14.8 ± 6.7	14.7 ± 8.5	14.8 ± 6.1	NS
Operation time (minutes)	89 [38–225]	127.5 [90–225]	77 [38–152]	<0.0001
Postoperative pain (NRS 0–10)				
Day 1	3 [2–6]	4 [3–5]	3 [2–6]	0.002
Day 2	3 [1–4]	3 [2–4]	2 [1–4]	0.003
Satisfaction with the surgery (n = 64)				
very satisfied	54 (84.4%)			
satisfied despite some discomfort	9 (14.1%)			
regret	1 (1.6%)			

Data are presented as median [range] or mean ± standard deviation. NS, not significant; NRS, numerical rating scale.

**Table 3:** Postoperative complications (n = 81).

Characteristics	Total Cases	Initial 20 Cases	Subsequent 61 Cases	*p* Value
Transient postoperative complications				
voiding difficulty	4 (4.9%)	1 (5.0%)	3 (4.9%)	NS
urinary frequency	5 (6.2%)	2 (10.0%)	3 (4.9%)	NS
urinary incontinence	3 (3.7%)	1 (5.0%)	2 (3.3%)	NS
wound infection	1 (1.2%)	0	1 (1.6%)	NS
vaginitis	1 (1.2%)	0	1 (1.6%)	NS
constipation	1 (1.2%)	0	1 (1.6%)	NS
perineal sensory abnormalities	1 (1.2%)	1 (5.0%)	0	NS
laceration of the suture site	1 (1.2%)	0	1 (1.6%)	NS
Total	17 (21.0%)	5 (25%)	12 (19.7%)	NS
Long-term complications				
Persistent urinary incontinence	1 (1.2%)	0	1 (1.6%)	NS
Recurrence of pelvic organ prolapse	3 (3.7%)	0	3 (4.9%)	NS
De novo rectal prolapse	2 (2.5%)	1 (5.0%)	1 (1.6%)	NS
Total	6 (7.4%)	1 (5.0%)	5 (8.2%)	NS

NS, not significant.

## Data Availability

The data presented in this study are available on request from the corresponding author. Research data can only be provided upon request due to individual privacy concerns.

## References

[1] Choi K.H., Hong J.Y. (2014). Management of pelvic organ prolapse. Korean J. Urol..

[2] Vergeldt T.F., Weemhoff M., IntHout J., Kluivers K.B. (2015). Risk factors for pelvic organ prolapse and its recurrence: A Systematic Review. Int. Urogynecol. J..

[3] Jelovsek J.E., Barber M.D. (2006). Women seeking treatment for advanced pelvic organ prolapse have decreased body image and quality of life. Am. J. Obstet. Gynecol..

[4] Department of Economic and Social Affairs of United Nations World Population Prospects 2024. https://population.un.org/wpp/graphs.

[5] Korean Statistical Information Service South Korea by Population 2024. https://kosis.kr/visual/populationKorea/PopulationDashBoardMain.do.

[6] Abbasy S., Kenton K. (2010). Obliterative procedures for pelvic organ prolapse. Clin. Obstet. Gynecol..

[7] Persu C., Chapple C.R., Cauni V., Gutue S., Geavlete P. (2011). Pelvic Organ Prolapse Quantification System (POP–Q)–A New Era in Pelvic Prolapse Staging. J. Med. Life.

[8] Park J.Y., Han S.J., Kim J.H., Chun K.C., Lee T.S. (2019). Le Fort partial colpocleisis as an effective treatment option for advanced apical prolapse in elderly women. Taiwan. J. Obstet. Gynecol..

[9] van der Vaart L.R., Vollebregt A., Milani A.L., Lagro-Janssen A.L., Duijnhoven R.G., Roovers J.-P.W., van der Vaart C.H. (2022). Effect of pessary vs surgery on patient-reported improvement in patients with symptomatic pelvic organ prolapse: A Randomized Clinical Trial. JAMA.

[10] de Albuquerque Coelho S.C., de Castro E.B., Juliato C.R.T. (2016). Female pelvic organ prolapse using pessaries: Systematic Review. Int. Urogynecol. J..

[11] Niigaki D.I., Silva R.S., Bortolini M.A.T., Fitz F.F., Castro R.A. (2022). Predictors for long-term adherence to vaginal pessary in pelvic organ prolapse: A Prospective Study. Int. Urogynecol. J..

[12] Koch M., Carlin G., Lange S., Umek W., Krall C., Bodner-Adler B. (2023). Long-term adherence to pessary use in women with pelvic organ prolapse: A Retrospective Cohort Study. Maturitas.

[13] Jones K., Wang G., Romano R., St Marie P., Harmanli O. (2017). Colpocleisis: A Survey of Current Practice Patterns. Urogynecology.

[14] FitzGerald M.P., Richter H.E., Siddique S., Thompson P., Zyczynski H., Ann Weber for the Pelvic Floor Disorders Network (2006). Colpocleisis: A Review. Int. Urogynecol.J..

[15] FitzGerald M.P., Richter H.E., Bradley C.S., Ye W., Visco A.C., Cundiff G.W., Zyczynski H.M., Fine P., Weber A.M. (2008). Pelvic support, pelvic symptoms, and patient satisfaction after colpocleisis. Int. Urogynecol. J..

[16] South M., Amundsen C. (2007). Overt rectal prolapse following repair of stage IV vaginal vault prolapse. Int. Urogynecol. J..

[17] von Pechmann W.S., Mutone M., Fyffe J., Hale D.S. (2003). Total colpocleisis with high levator plication for the treatment of advanced pelvic organ prolapse. Am. J. Obstet. Gynecol..

[18] Shull B.L., Capen C.V., Riggs M.W., Kuehl T.J. (1992). Preoperative and postoperative analysis of site-specific pelvic support defects in 81 women treated with sacrospinous ligament suspension and pelvic reconstruction. Am. J. Obstet. Gynecol..

[19] O’Leary A.J., Vyas S.K. (2004). Le Fort’s partial colpocleisis: A review of one surgeon’s experience. Gynecol Surg..

[20] Keskin D.D., Keskin S. (2023). Le Fort Partial Colpocleisis: An Early and Feasible Option in Pelvic Organ Prolapse. Parity.

[21] Cohn J.A., Smith A.L. (2019). Management of occult urinary incontinence with prolapse surgery. Curr. Urol. Rep..

[22] Sung V.W., Weitzen S., Sokol E.R., Rardin C.R., Myers D.L. (2006). Effect of patient age on increasing morbidity and mortality following urogynecologic surgery. Am. J. Obstet. Gynecol..

[23] Song X., Zhu L., Ding J., Xu T., Lang J. (2016). Long-term follow-up after LeFort colpocleisis: Patient Satisfaction, Regret Rate, and Pelvic Symptoms. Menopause.

[24] Zebede S., Smith A.L., Plowright L.N., Hegde A., Aguilar V.C., Davila G.W. (2013). Obliterative LeFort colpocleisis in a large group of elderly women. Obstet. Gynecol..

